# The transport of a charged peptide through carbon nanotubes under an external electric field: a molecular dynamics simulation

**DOI:** 10.1039/d0ra09184g

**Published:** 2021-07-05

**Authors:** Wen Li, Shun Cheng, Bin Wang, Zheng Mao, Jianhua Zhang, Youyu Zhang, Qing Huo Liu

**Affiliations:** Institute of Electromagnetics and Acoustics, and Department of Electronic Science, Xiamen University Xiamen 361005 P. R. China jhzh@xmu.edu.cn; Department of Electrical and Computer Engineering, Duke University Durham NC 27708 USA qhliu@duke.edu; Shenzhen Research Institute of Xiamen University Xiamen 361005 P. R. China 409593818@qq.com; Nanjing Institute of Technology No.1 Hongjing Avenue of Jiangning District Nanjing 211167 China zhengmao_xmu@outlook.com; Department of Physics, Hainan University 570228 Hainkou P. R. China jianhuazhang@hainanu.edu.cn

## Abstract

The study of interactions between biomolecules and carbon nanotubes (CNTs) is of great importance in CNT-based drug delivery systems and biomedical devices. In this work, the transport of polyarginine (R8) peptide through CNTs under an external electric field was investigated *via* all-atom molecular dynamics (AAMD) simulation. It was found that the electric field can assist the R8 peptide to overcome the resistance and make the transport smooth. Moreover, the efficiency of transport was improved with the increasing intensity of the electric field in a suitable range. In addition, we also investigated the effects of different types of CNTs on the transport of the R8 peptide and found that the single-walled carbon nanotube (SWCNT) was more suitable for transporting the R8 peptide than the double-walled carbon nanotube (DWCNT) due to its lower energy barrier to the R8 peptide. All these findings shed light on the role of the electric field on the transport of the R8 peptide through CNTs and also gave some valuable insights into the effects of CNT types on the transport process of the peptide.

## Abstract 2

The role of electric field and types of carbon nanotube influencing the delivery process of peptide through CNTs were studied *via* all-atom molecular dynamics simulation.

## Graphical abstract

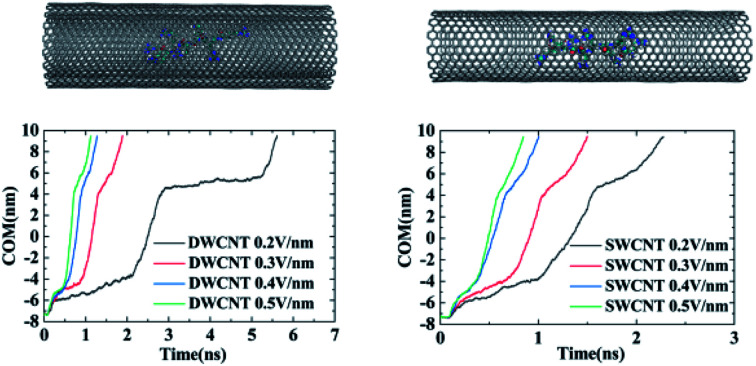

## Introduction

The cell-penetrating peptides (CPPs) have been widely studied as a drug carrier due to their advantages of high transport efficiency, low cytotoxicity, and ease of molecular design.^1^ Several CPP-based treatments have already entered clinical trials.^2–4^ Among these CPPs, the R8 peptide has recently attracted significant attention as a drug carrier because of its ability to cross cell membranes alone or with cargo.^5,6^

On the other hand, CNTs have also caught the eyes of researchers in various fields for their intrinsic structure^7^ and desirable properties.^8^ The open-ended CNT has a hollow cylindrical structure and consists of rolled graphite sheets with carbon atoms as a backbone. Due to the ability to encapsulate different kinds of molecules, CNTs have been proven as one of the excellent transport candidates for encapsulating and delivering many molecules. For instance, HRIP,^9^ SmtA,^10^ and RNA^11^ have been studied previously, and the results indicated that these molecules could be spontaneously encapsulated into the CNTs. Furthermore, numerous applications of CNTs have been reported in physical, biotechnological, and biomedical fields, such as hydrogen storage,^12–14^ desalination,^15^ fullerenes encapsulation^16,17^ biosensors,^18,19^ bio-catalysts,^20^ and biomedical devices.^21,22^

Although previous experiments and simulations greatly enhanced our knowledge on the effects of CNTs on the transport of biomolecules,^9,23–25^ the effects of the controllable external tools such as magnetic or electrical forces on the transport of nanoparticles (NPs) still have received rare attention.

In particular, Q. Chen reveals that the CNTs can trap peptide drugs, and explain the influence of different diameter of CNTs on drug delivery devices,^41^ S. U. Lee analyzes the electron transport characterizes between CNTs and peptide linkages,^42^ Wang *et al.* reveal that an external electric field can speed up the transport process of the small mastoparan-X peptide through the SWCNT,^26^ and Francesco Puoci *et al.* also confirm that the application of an external electric field can enhance the delivery of diclofenac.^27^ Herein, we attempt to investigate the effects of an external electric field on the transport of R8 peptide through CNTs from the respect of the first-principle simulation, to find out whether the external electric field would affect the transport process of R8 peptide and the mechanisms involved in.

In the previous works, MD simulations were considered as one of the powerful approaches in exploring biomolecule–CNTs interactions.^28,29^ The dynamic mechanism of CNTs with other molecules can be systematically studied and further investigated at the molecular level by using MD simulations. Hence the AAMD simulations were employed in this work to provide an in-depth understanding of the interactions and the dynamic mechanisms of the R8-CNTs systems. Two systems, including R8-SWCNT and R8-DWCNT systems, were constructed using AAMD models. The transport of R8 peptide through CNTs was simulated and analyzed with or without the action of an external electric field. Results obtained from the present work provide not only an insight into the role of an electric field in prompting peptide drug (or drug complex based on peptide carrier) delivery but also some new knowledge into the different effects of types of the CNTs (single-walled and double-walled) on the transport of R8 peptide.

## Methods and models

### 1. CNT models

**Fig. 1:**
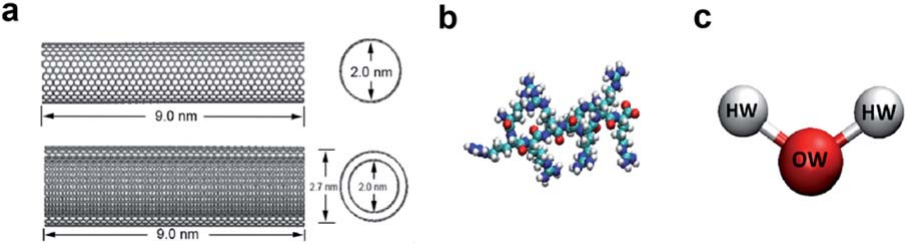
(a) The structures of SWCNT (upper) and DWCNT (lower). (b) The structure of a single all-atom R8 peptide. (c) The SPCE water model.

In the model systems, the armchair (15, 15) SWCNT (see Fig. 1a, upper part) was constructed with a length of 9.0 nm and a diameter of 2.0 nm. Likewise, the (15, 15) DWCNT (see Fig. 1a, lower part) has the same length as SWCNT with 2.0 nm diameter of its inner tube and 2.7 nm of the outer tube. The structures of the CNTs were constructed with a C–C bond with an equilibrium distance of 1.42 Å and an equilibrium angle *Θ*_0_ = 120°.

### 2. The R8 peptide model

The R8 peptide, with an average diameter of 1.6 nm in the simulations, was constructed based on the OPLS-AA force field and comprised of eight arginine amino acids (see Fig. 1b), which results in the R8 peptide bearing eight positive charges. The bond, angle, and dihedral potential energy functions were used to represent the bonded interactions, while the Lennard–Jones (L–J) interaction and Coulomb energy functions were applied to describe non-bonded interactions in the simulation.^43,44^

### 3. The R8-CNTs systems and simulation details

**Fig. 2:**
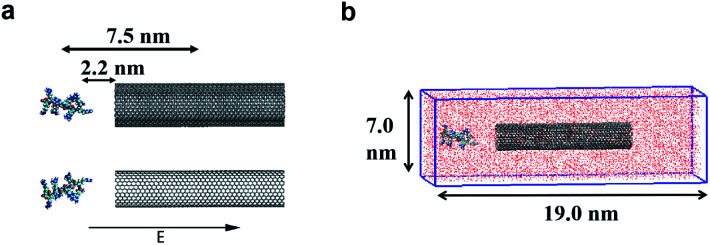
(a) The models of the R8-CNTs systems with an external electric field aligned along the CNTs. (b) The simulation box with the R8-CNTs models embedded in a periodic box with dimensions 7 nm × 19 nm × 7 nm, the red particles full of the box represent the water solvent molecules.

In the present work, two systems, including the R8-SWCNT and R8-DWCNT systems were constructed (see Fig. 2). The R8 peptide was initially placed near one side of the CNTs, and the centre of mass (COM) distance between the peptide and the CNTs was 7.5 nm, indicating that the initial distance between the periphery of the peptide and the CNTs was about 2.2 nm (bigger than the cut-off distance), so that there is no interaction energy between them initially. The whole system was embedded in a water box with dimensions 7.0 nm × 19.0 nm × 7.0 nm, and periodic boundary conditions were applied for all sides of the box, the MD simulations were performed with the OPLS-AA force field for CNTs and R8 peptide. Flexible SPCE water model (see Fig. 1c)^30,31^ was used as a solvent in all systems. Moreover, eight chloride ions were added into the simulation box to neutralize the positive charges of the R8 peptide. The tubes were aligned along the *Y* direction and fixed in the simulations.

Besides, the V-rescale method was employed to control the system temperature at 298.15 K,^32,33^ and the pressure of 1 bar was coupled using the Parrinello–Rahman barostat.^34^ The cut-off method with a cut-off distance of 16 Å was used to calculate the van der Waals (VDW) interactions, and the Particle Mesh Ewald (PME)^35^ summation with a grid size of 16 Å was used for calculating long-range electrostatic (Coulomb) interactions. The non-bonded interaction was described by the VDW interaction combining with the Coulomb interaction. The time-step was 2 fs (ref. 14 and 28) in all simulations. Systems were minimized and equilibrated for 2 ns at 298.15 K using the isothermal–isobaric (NPT) ensemble^36^ with a time step of 1 fs at the initial stage of simulation.

For the cases with an electric field, the external electric fields were added to the two systems along the positive *Y* direction. Two ranges of electronic intensity are considered in the present work: one is from 0.04 V nm^−1^ to 0.09V nm^−1^ with an interval of 0.01 V nm^−1^ and the other is from 0.1 V nm^−1^ to 0.5 V nm^−1^ with an interval of 0.1 V nm^−1^. For each intensity of field ranging from 0.04 V nm^−1^ to 0.09 V nm^−1^, it runs at least 3 times and each run lasts no less than 30 ns long. In total, there are 30 times runs and 41 runs for both the cases of SWCNT and DWCNT, for each intensity of another filed ranging from 0.1 V nm^−1^ to 0.5 V nm^−1^, it runs at least 3 times and each run lasts no less than 7 ns long. In total, there are 15 times runs and 20 times runs for both the cases of SWCNT and DWCNT.

The applied electric field was taken into a driving force *F* = *q*_*i*_*E* for all beads with a charge *q*_*i*_, including the positively charged R8 peptide, water model beads, and other charged beads in all systems. The data were collected every 1 ps. The COM distance between the peptide and CNTs was used as the reference point for the matter movement.

RMSD is an important basis for measuring the stability of the system group and it is dominated by the following function:
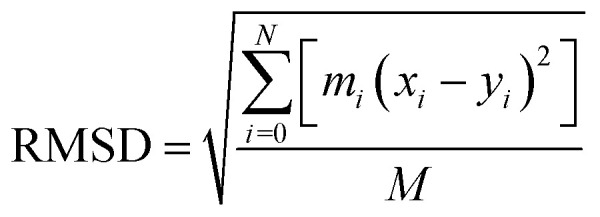
where *N* is the number of atoms, *m*_*i*_ is the mass of atom *i*, *x*_*i*_ is the coordinate vector for target atom *i*, *y*_*i*_ is the coordinate vector for reference atom *i*, and *M* is the total mass. If the RMSD is not mass-weighted, all *m*_*i*_ = 1 and *M* = *N*. When calculating the RMSD of a target to a reference structure, not only does the number of atoms but also the atomic arrangement in the target was required to match that in the reference. The changes of RMSD were also analyzed in the present work.

To assess the energy cost of the R8 peptide through the CNTs, the free energy profiles of the penetration were extracted in the form of a 1D potential mean force (PMF) profile. In the present work, PMF profiles were calculated from the standard combination of the umbrella sampling protocol^37^ and the implementation of the weighted histogram analysis method (WHAM).^38^ Pull simulations were performed over a distance of 16.0 nm along the axial direction of the CNTs by applying a constant force of 1000 kJ mol^−1^ nm^−2^ (ref. 37) and a constant velocity of 0.01 nm ps^−1^. The selected configurations with a regular spacing of 0.2 nm extracted from the trajectory files of the pulling process were used as the input windows of umbrella sampling.

All simulations in the present work were performed using the Gromacs 4.5.5 (ref. 39) program and results were visualized by Visual Molecular Dynamics (VMD) software.^40^

## Results and discussions

### 1. Free energy profiles

**Fig. 3:**
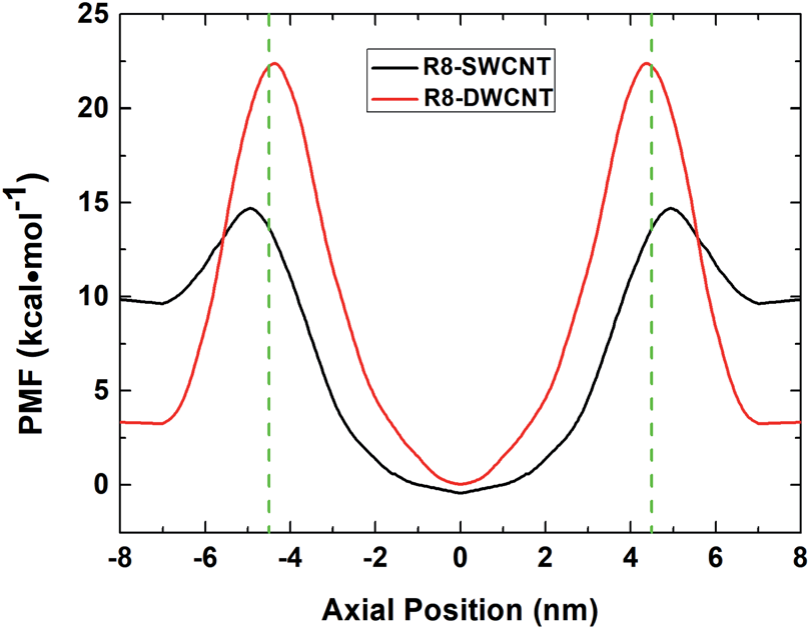
The PMF profiles of R8 peptide passing through the SWCNT (black line) and the DWCNT (red line). The green dash lines indicate the position of the two sides of the CNTs.

The free energy profiles (see Fig. 3) of the R8-CNTs system were analyzed to understand the difference of R8 peptide transporting through the two types of CNTs, for the ability of R8 peptide transporting through the CNTs mainly depends on the level of the energy barrier. In the present work. The constant pulling velocity was set at 0.01 nm ps^−1^, the horizontal coordinate −4.5 nm and 4.5 nm (green dash lines in Fig. 3) represent both sides of the CNTs, and the horizontal coordinate 0 means the middle of the CNTs. The whole PMF curves could be divided into three stages (taking the R8-DWCNT case for example): (i) firstly, as the R8 peptide moving close to the entrance of the DWCNT, the free energy first decreased a bit (from 3.33 kcal mol^−1^ to 3.23 kcal mol^−1^) and thus produced a very small local minimum energy point (at ±7.0 nm), which meant that the R8 peptide and the DWCNT were slightly attracting each other, and then the energy barrier swiftly increased when the R8 peptide continued drawing on (−7 nm to −4.5 nm); such a high resistance might come from the break of the hydrogen bond networks around the peptide and repelling the water molecules inside or outside of the CNT during the transport process. (ii) After passing through the entrance of the DWCNT, the PMF exhibited a significant reduction (from 22.38 kcal mol^−1^ to 0 kcal mol^−1^) of energy barrier until the R8 peptide reached the center of the DWCNT, and then the energy barrier rose rapidly again (0 nm to +4.5 nm) when the R8 peptide approaching the exit of the DWCNT. Notably, at that moment, the L–J attraction between the peptide and the DWCNT had turned into resistance for hindering the exiting of the peptide. (iii) When the R8 peptide came out of the DWCNT, the barrier decreased again (+4.5 nm to +7 nm), and produced a similar small local energy minimum point (at about +7 nm) near the exit of the DWCNT.

Likewise, it exhibited similar changes in the PMF profile when the R8 peptide passing through the SWCNT. The energy barrier was noticeably lower than passing through the DWCNT (5.08 kcal mol^−1^*vs.* 19.15 kcal mol^−1^), which suggested that the R8 peptide was easier to transport through SWCNT than DWCNT.

### 2. Interaction of R8 peptide with CNTs in the absence of the external electric field

To investigate the interaction of the R8 peptide with CNTs, the two systems were performed for 100 ns. It was found that the spontaneous transports of the R8 peptide through CNTs were not observed without an applied electric field. The R8 peptide seemed to prefer to stay near the side of the CNTs through the whole simulation rather than get into the CNTs. This phenomenon might be caused by the hydrophilic properties of the R8 peptide.

### 3. Interaction of R8 peptide with CNTs in the presence of an external electric field ranging from 0.01 V nm^−1^ to 0.09 V nm^−1^

**Fig. 4:**
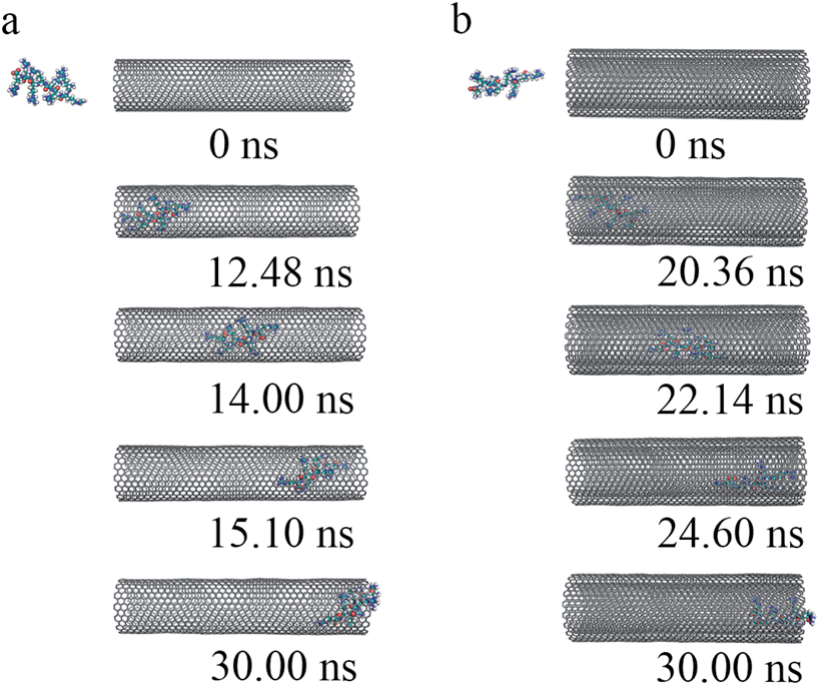
The key snapshots of the transport of R8 peptide through the SWCNT (a) and the DWCNT (b) under an external electric field (0.05 V nm^−1^).

**Fig. 5:**
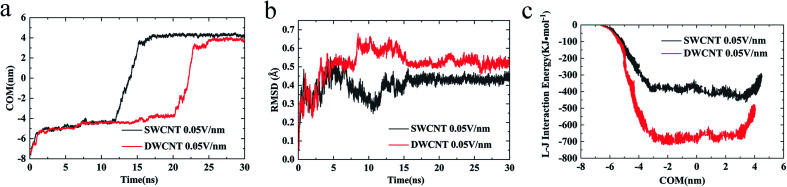
The COM distance between the R8 peptide and CNTs (a) and the RMSD of R8 peptide in the transport (b) with a 0.05 V nm^−1^ external electric field. The L–J (van der Waals) interaction energy of R8 peptide in the transport under a 0.05 V nm^−1^ external electric field (c).

(i) To improve the efficiency of R8 peptide transport through carbon nanotubes, a feasible method is to add an electric field along the direction of the nanotube since the R8 peptide is charged. A driving force is introduced by the applied electric field, which can increase the probability of the peptide penetrating through the CNTs. Firstly, we examined the effect of the external electric field ranging from 0.01 V nm^−1^ to 0.09 V nm^−1^ on the R8 peptide penetration process. In these simulations, the R8 peptide was found to have a certain probability of getting into the CNTs if the intensity of the electric field is not less than 0.04 V nm^−1^, but it cannot get out of the CNTs during the whole simulation time. The probabilities of R8 peptides entering SWCNT and DWCNT are 36.67% and 29.27%, respectively. This is consistent with the PMF calculations. As shown above, the energy barrier for R8 entering DWCNT is higher than the one for R8 peptide getting into SWCNT. The higher the energy barrier, the lower the entering probability.

(ii) We are also interested in the effects of the electric field on the transport of R8 peptide in CNTs. For this reason, we examined the time that it takes R8 peptide to travel from one end of the CNTs to the other (−3 nm to 3 nm in *Y*-axis) under the electric field with different intensities. The average times required for R8 peptide transport in the cases of SWCNT and DWCNT are 2.063 ns and 2.307 ns, respectively. We could see that the R8 peptide runs faster in SWCNT than in DWCNT under the same ranges of electric intensity.

(iii) To get a detailed understanding of the process of R8 penetration into CNTs, we took one example from each of the cases of R8-SWCNT and R8-DWCNT systems under an external field of 0.05 V nm^−1^. The key snapshots of the transport of R8 peptide into SWCNT and DWCNT were shown in Fig. 4a and b. It took about 12.48 ns for the R8 peptide to enter the SWCNT. Then the R8 peptide continued to travel from one end of the SWCNT to the other (−3 nm to 3 nm in *Y*-axis) quickly (within 2.62 ns). However, the R8 peptide cannot get out of the SWCNT but got stuck at the outlet of SWCNT. Finally, it oscillated at the SWCNT outlet until the end of the simulation. Likewise, we could see that it took about 20.36 ns for R8 peptide to enter the DWCNT, which was twice as much as that in the SWCNT case, then it took about 4.24 ns to travel from one end of the DWCNTs to the other, which was longer than that spent in the SWCNT case. Similarly, it eventually oscillated at the outlet of the DWCNT.

(iv) The changes of COM distance between the R8 peptide and CNTs were exactly corresponding to the R8's three different stages (shown in Fig. 5a): entering CNTs, passing through CNTs, and oscillating at the outlet of the CNTs. But it took more time for R8 peptide to enter DWCNT than SWCNT, probably because that the energy barrier for R8 entering DWCNT is higher than the one for R8 getting into SWCNT. As shown in Fig. 5b, the RMSD increased significantly at the beginning, and then remained stable with time elapse. We could see that the two systems have reached a stable state after running for 30 ns. As for the VDW (L–J) interaction energy between R8 and CNTs along with the *Y*-axis, we found that the absolute value of the L–J interaction energy of R8 with CNT in the process of the R8 peptide penetration into the SWCNT always smaller than that in the process of the R8 peptide penetration into the DWCNT (see Fig. 5c), especially when R8 peptide was inside the CNTs. It means that the attraction between R8 peptide and DWCNT is stronger than the one between R8 peptide and SWCNT. It may be one of the reasons that the R8 peptide penetrated slower in DWCNT than in SWCNT. Besides, the R8 peptide cannot come out of the CNTs but in a state of oscillation close to the outlet of CNTs, which may because the electric field is too small that R8 peptide is difficult to overcome the energy barriers.

### 4. Interaction of R8 peptide with CNTs in the presence of an external electric field ranging from 0.1 V nm^−1^ to 0.5 V nm^−1^

#### 4.1 The transport of R8 peptide through CNTs

**Fig. 6:**
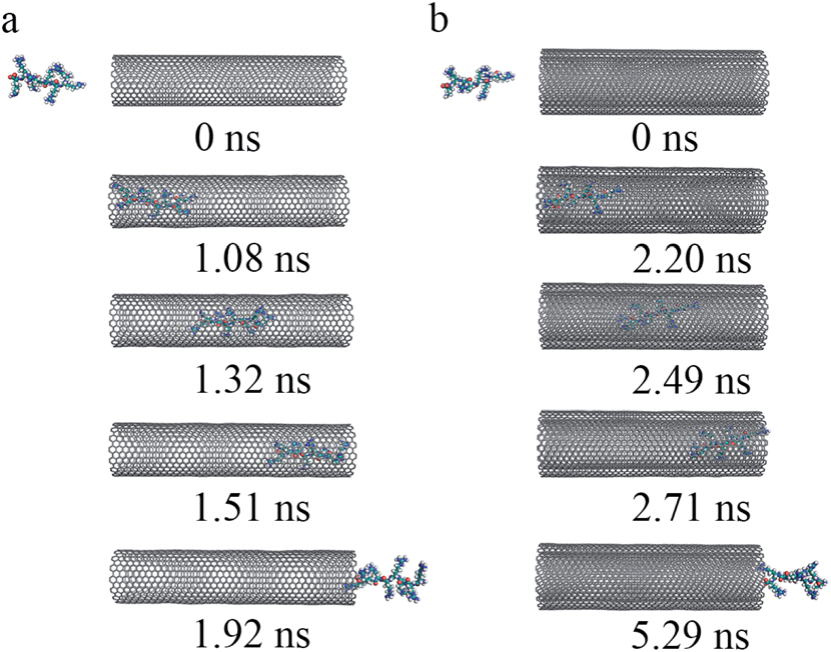
The key snapshots of the transport of R8 peptide through the SWCNT (a) and the DWCNT (b) under an external electric field (0.2 V nm^−1^).

As shown above, the R8 peptide only can enter into the CNTs but cannot get out of the CNTs if the external electric intensity is on the order of 0.01 V nm^−1^. To further insights into the effects of external electric fields on the process of R8 peptide penetration through CNTs, we increased the intensity of external electric fields (ranging from 0.1 V nm^−1^ to 0.5 V nm^−1^) to examine the potential effects on the R8 peptide penetration process. In our simulations, it was found that the R8 peptide can penetrate through the CNTs if the intensity of the electric field is not less than 0.2 V nm^−1^. An external electric field of 0.2 V nm^−1^ in a water solvent environment was applied to the R8-CNTs systems. The key snapshots of the transport of the R8 peptide through SWCNT and DWCNT were shown in Fig. 6a and b. The R8 peptide was entering the CNTs within 1.08 ns for SWCNT and 2.20 ns for DWCNT, then moved on in CNTs rapidly, and finally came out of the whole CNTs at 1.92 ns for SWCNT and 5.29 ns for DWCNT. The time it takes to transport R8 peptide through DWCNT (about 5.29 ns) was longer than that in the SWCNT case (1.92 ns).

#### 4.2 The comparison of the two transport processes

**Fig. 7:**
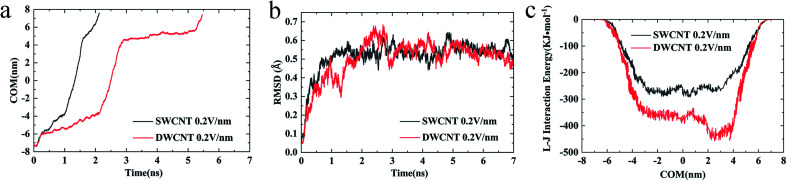
(a) The COM distance between the R8 peptide and CNTs, (b) the RMSD of R8 peptide, and (c) the L–J (van der Waals) interaction energy of R8 peptide and CNTs in the transport under an external electric field of 0.2 V nm^−1^.

The transport processes of R8-SWCNT and R8-DWCNT systems under an electric field (0.2 V nm^−1^) were further analyzed *via* the COM distance, RMSD (root mean square deviation), and VDW interaction energy. As shown in Fig. 7a, the profiles of the COM distance of both systems were similar to the cases under the electric field (0.05 V nm^−1^), the difference was that COM distances under an electric field (0.2 V nm^−1^) became larger than 4.5 nm at the end of the simulations because the R8 peptide finally came out of the CNT for both systems. Comparing with the R8-SWCNT system, the R8-DWCNT system took more time during the entering and the exiting processes. And as shown in Fig. 7b, the RMSD value has relatively large fluctuations during the entering and the exiting processes, then stabilized at about 0.5 Å. the L–J interaction energy along the *Y*-axis between R8 peptide and CNTs are finally analyzed (see Fig. 7c). It was found that the absolute value of the L–J interaction energy between R8 peptide and CNTs during the R8 peptide penetration process under an electric field of 0.2 V nm^−1^ was smaller than that under an electric field of 0.05 V nm^−1^. The value of the L–J interaction energy of R8 and SWCNT was always smaller than that of R8 and DWCNT during the whole R8 penetration process.

**Fig. 8:**
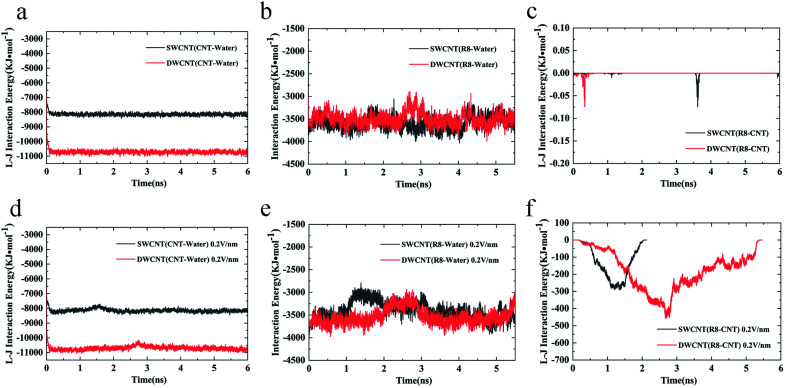
The L–J (van der Waals) interaction energy of (a) CNTs and water, and (c) R8 peptide and CNTs under an external electric field of 0 V nm^−1^. The L–J (van der Waals) interaction energy of (d) CNTs and water, and (f) R8 peptide and CNTs under an external electric field of 0.2 V nm^−1^. The interaction energy of R8 peptide and water under an external field of (b) 0 V nm^−1^ and (e) 0.2 V nm^−1^.

Considering the influence of the interaction of different molecules (water, CNTs, and R8 peptide) on the transport process, we also analyzed the variations of the interaction energy (including L–J interaction energy and the Coulomb interaction energy) between them during the simulation time under the electric field of 0 V nm^−1^ and 0.2 V nm^−1^. It should be noted that the Coulomb interaction energies between CNTs and other molecules are zero because the CNTs have no charges in the present model. As shown in Fig. 8a and d, it was found that the value of the L–J interaction energy of CNTs and water was almost the same for both systems under an electric field of 0 V nm^−1^ and 0.2 V nm^−1^. But under the two different electric field exposure, the transport state of the R8 peptide changed obviously. The R8 peptide was able to get through the CNTs under the 0.2 V nm^−1^ whereas it could not happen in the simulation without an external electric field. Thus, we presumably thought that the interaction between CNTs and water may have little influence on the delivery of the R8 peptide through CNTs. The interaction energy between R8 peptide and water under the electric field of 0 V nm^−1^ and 0.2 V nm^−1^ are shown in Fig. 8b and e. For the case of 0 V nm^−1^, the interaction energy between the R8 peptide and water fluctuated around a certain value of about 3535 KJ mol^−1^ during the whole simulation time. However, for the case of 0.2 V nm^−1^, the interaction energy of the R8 peptide with water decreased when the R8 peptide moved into the CNTs and increased when the R8 peptide came out of the CNTs. This may be because the number of water molecules around the R8 peptide in the CNTs is smaller than that out of the CNTs. Therefore, the attraction from water to the R8 peptide out of the CNTs is stronger than that in the CNTs. It could be one of the reasons that the transport of R8 peptide in the CNTs was easier than that in pure water. As shown in Fig. 8c, without the external electric field, the L–J interaction energy of R8 peptide and CNTs was so small that it was almost equal to 0. This might due to that without the pull of the electric field force, the R8 peptide stayed outside the CNTs during the whole simulation time which leads to low attractions. However, the L–J interaction energy of R8 peptide and CNTs changed significantly with the electric field of 0.2 V m^−1^ (see Fig. 8f), showing relatively strong interactions between R8 peptide and CNTs, under which the R8 peptide ultimately get through the CNTs. Thus, it was conclusively supposed that the transport of R8 peptide was notably affected by the electric field applied, and the attractions from CNTs and water. Besides, it was found that under an electric field of 0.2 V nm^−1^ the total interaction energy of the R8 peptide with the other molecules (CNT and water) in the transport process of R8 peptide through SWCNT was smaller than that through DWCNT. This might be the major reason that the R8 peptide needs to take more time to penetrate the DWCNT than to penetrate the SWCNT.

Based on these results, we could further analyze the difference between R8-CNTs cases. During the period of R8 peptide entering the CNTs, the COM distances between R8 peptide and CNTs deceased with a small gradient, while the L–J interaction energies of the R8 peptide with CNTs exhibited sustaining decrease. The major reason for these changes might be the resistance contributed by the break of the hydrogen bond networks of the water molecules around the peptide by the attraction of CNTs when it moved close to the entrance of the CNTs. While the COM distance decreased fastly when the R8 peptide was moving on inside the CNTs, the L–J interaction energy was almost unchanged, indicating that the transport resistance reduced when the R8 peptide was inside the CNTs, the R8 peptide was droved to move forward quickly under an electric field, and thus speed up the decrease of the COM distance. Similarly, the R8 peptide had to overcome another resistance during the exporting period.

### 5. Effects of electric field strength on the transport of R8 peptide in CNTs

**Fig. 9:**
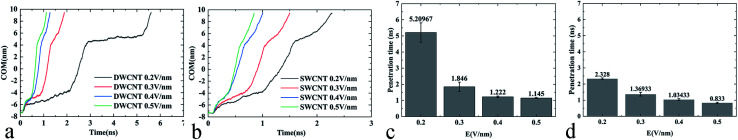
(a) The COM distance between the R8 peptide and the DWCNT as a function of simulation time under various external electric fields. (b) The COM distance between the R8 peptide and the SWCNT as a function of simulation time under various external electric fields. (c) The average transport time of R8 peptide through the DWCNT under various electric fields. (d) The average transport time of R8 peptide through the SWCNT under various electric fields.

External electric fields of different intensities were applied as a powerful and controllable means to prompt the transport of R8 peptide in the present work, and the results showed that the electric field could indeed prompt the transport process of the R8 peptide in CNTs (see Fig. 9):

(i) The changes of COM distance (Fig. 9a and b) were all similar to the effects of the individual strength of the external electric field. When the R8 peptide moved to the entrance, the COM distance in all cases decreased with a small gradient, but swiftly decreased with a bigger gradient as the R8 peptide entered into the SWCNT, and then it decreased slowly again when the R8 peptide moved out of the SWCNT. However, the rate of the decline during each stage changed in a dose-dependent manner with the electric field strength. And we could see that the tendency of the COM distance for the R8-DWCNT system was the same as that for the R8-SWCNT system.

(ii) In Fig. 9c and d, the means and standard deviations of means (SD) of the penetration time were calculated from individual values, and factorial ANOVA followed by the independent-samples *T*-test *via* SPSS 13.0 were used to determine the statistical significance. Data are presented as mean ± S.D., and differences were considered significant at *p* < 0.05. It was found that the penetration time was decreased evidently when the strength of the electric field increased to 0.3 V nm^−1^, then the penetration time was slowly decreased as the electric field strength further increased.

Throughout the whole transport process under various electric field intensities, it was found that the electric field could promote the movement of the R8 peptide in a dose-dependent manner. However, when the strength of the applied electric field was strong enough, the decrease of the penetration time was not obvious anymore, and finally, the time cost of the penetration would stabilize at a certain small value, which indicated that the transport had been able to achieve efficiently under such strong electric fields (stronger than 0.3 V nm^−1^).

## Conclusions

In the present work, the transport of R8 peptide through CNTs with various intensities of external electric fields had been investigated using AAMD simulations. Firstly, it was found that R8 peptide cannot spontaneously transport through the CNTs and stay near the entrance of the CNTs in the absence of an external electric field, which meant that as a hydrophilic peptide, the R8 peptide preferred to stay into the water solvent rather than getting inside the CNTs. This phenomenon could be explained by the PMF profile, when the R8 peptide moved close to the entrance of the CNTs, a high energy barrier existed to hinder the transport. Next, when introducing external electric fields (ranging from 0.01 V nm^−1^ to 0.09 V nm^−1^) to the systems, the transport of R8 peptide could be observed within 30 ns, the electric field force became the dominant driving force for the movement of R8 peptide, while the L–J attraction force between the R8 peptide and the CNTs also played a vital role in the transport. And we observed that the R8 peptide cannot come out of the CNTs but in a state of oscillation close to the outlet of CNTs, which may be because the electric field is too small that R8 peptide is difficult to overcome the energy barriers. Although an energy barrier existed in both sides of the CNTs (see Fig. 3) to hinder the transport of peptide, but when we introduced external fields (ranging from 0.1 V nm^−1^ to 0.5 V nm^−1^) when the external field increased no less than 0.2 V nm^−1^, the driving force came from an external electric field helped the R8 peptide to easily overcome the resistance and made the transport smoothly within a few nanoseconds. In other words, an external electric field could indeed be applied as an effective tool to promote the transport of the R8 peptide. Moreover, it was also found that the transport efficiency of the R8 peptide was improved gradually in pace with the improvement of the electric field strength (0.2 V nm^−1^ to 0.5 V nm^−1^).

On the other hand, it was found that the R8 peptide would cost more time to transport through the DWCNT than through the SWCNT. This phenomenon also could be explained in terms of the energy barrier: when moving through the entrance or the exit of the DWCNT, the R8 peptide had to overcome a higher energy barrier than through the SWCNT. Consequently, a larger time cost was required to accomplish the transport process. Thus we could conclude that the SWCNT was more suitable for transporting R8 peptide than the DWCNT in terms of transport efficiency.

## Conflicts of interest

There are no conflicts to declare.

## Supplementary Material
